# A Case of Cala Trio-Induced Glossopharyngeal Neuralgia in a Patient with Essential Tremor and Idiopathic Parkinson’s Disease

**DOI:** 10.5334/tohm.1224

**Published:** 2026-06-15

**Authors:** Moriah R. Arnold, Amie L. Hiller

**Affiliations:** 1 Medical Scientist Training Program, Oregon Health and Science University, Portland, OR, USA; 2 Neurology Department, Oregon Health and Science University, Portland, OR, USA; 3 VA PADRECC –Portland Health Care System, Portland, OR, USA

**Keywords:** Essential tremor, Transcutaneous afferent patterned stimulation, Glossopharyngeal neuralgia, Neuromodulation, Parkinson’s Disease

## Abstract

**Background:**

Cala Trio is a wrist-worn transcutaneous afferent patterned stimulation (TAPS) device for essential tremor (ET), delivering high-frequency stimulation to the median and radial nerves.

**Case Report:**

We report the first case of glossopharyngeal neuralgia (GPN) associated with Cala Trio initiation in a 69-year-old male with ET and Parkinson’s disease, with prior GPN. Throat spasms triggered by swallowing and coughing began after initiation on two trials and resolved with discontinuation; evaluation was unrevealing.

**Discussion:**

We review TAPS mechanisms, GPN neuroanatomy, and circuit-level links between peripheral nerve stimulation and brainstem cranial nerve hyperexcitability, highlighting a novel adverse event and need for caution in patients with prior GPN.

**Highlights:**

We report the first case of glossopharyngeal neuralgia associated with Cala Trio TAPS therapy in a patient with essential tremor and Parkinson’s disease. Symptoms were temporally linked to device use and resolved upon discontinuation, suggesting a reversible neuromodulation-related effect and expanding the safety profile of peripheral tremor therapies.

## Introduction

Essential tremor (ET) is the most common movement disorder worldwide and is associated with substantial functional impairment and reduced quality of life [1,2,3,4,5]. Although traditionally considered a monosymptomatic action tremor, accumulating pathophysiologic evidence supports involvement of the cerebello-thalamo-cortical network, with abnormal oscillatory activity believed to originate within cerebellar circuitry, including Purkinje cell dysfunction [6,7]. While central network pathology is fundamental to ET, peripheral mechanisms also contribute to tremor expression. Sensory feedback loops, limb biomechanics, and reflex pathways between muscle spindles and spinal motor neurons influence tremor amplitude and frequency [8,9,10,11,12,13]. This dual central-peripheral framework has prompted investigation into neuromodulatory strategies, which target afferent pathways. Specifically, transcutaneous afferent patterned stimulation (TAPS) has emerged as a noninvasive therapeutic approach for upper limb tremor [14].

The Cala Trio system is a wrist-worn device designed to deliver alternating electrical pulses to the median and radial nerves at a frequency individualized to the patient’s tremor [15]. Clinical trials evaluating early iterations of the Cala device have reported improvements in tremor severity and activities of daily living with an overall favorable safety profile [16,17,18,19,20]. The conceptual basis of this therapy rests on activation of large-diameter sensory afferents, which transmit patterned input centrally and are hypothesized to influence tremor-related circuitry [14,15]. Preclinical and intraoperative neurophysiologic studies demonstrate that electrical stimulation of the median nerve at sub-motor threshold intensities evokes high-frequency oscillatory activity within the ventral intermediate nucleus of the thalamus implicated in tremor generation [15,21,22,23]. Additionally, stimulation-related responses have been observed in the subthalamic nucleus, likely reflecting propagation along medial lemniscal or thalamic projections [22].

Although transcutaneous afferent patterned stimulation is intended to modulate tremor-related networks and is emphasized in explaining therapeutic benefit, the full extent of its effects on interconnected central sensory pathways remains incompletely characterized. Peripheral afferent stimulation engages ascending projections that interface with multiple subcortical and brainstem structures, and as neuromodulatory therapies increasingly target distributed neural circuits, uncommon or unexpected neurologic effects may emerge as a side-effect profile. Cranial neuropathic pain syndromes, though rare, represent one potential manifestation of altered sensory network excitability. Among these, glossopharyngeal neuralgia (GPN) is characterized by brief, severe paroxysms of pain within the sensory territory of the glossopharyngeal nerve [24]. In this context, we report a patient with longstanding ET and newly diagnosed PD who had one episode of self-resolved GPN which returned after initiating Cala Trio therapy on two separate occasions. To our knowledge, this is the first reported association between TAPS and GPN.

## Case Description

The subject initially presented to the OHSU Movement Disorders Clinic as a 69-year-old male with a 20-year history of ET involving the bilateral upper extremities and a more recent 1-year history of resting tremor, balance changes, constipation, REM sleep behavior disorder, and hypophonia/hoarseness. Initial neurological examination demonstrated both action and resting tremor with mild parkinsonian features, including increased tone and bradykinesia in bilateral lower extremities and a slight postural stoop. Overall, the patient scored a 31/84 on the TRG ET performance rating assessment scale and 35 on the UPDRSm. A DaT scan revealed abnormal I-123 signal reflecting striatal dopamine depletion, ultimately leading to a diagnosis of idiopathic PD. Genetic testing subsequently identified a heterozygous E326K *GBA1* variant. The patient was started on levodopa with modest benefit, however continued to experience bothersome tremor attributable to a mixed ET/PD phenotype.

Past medical history was notable for hypertension secondary to white coat syndrome, GERD, hyperlipidemia, cluster headaches, ascending aortic aneurysm, and inguinal hernia. The patient’s cluster headaches were during a 3-year period 40 years prior and recurred episodically every several years, with complete symptom resolution between attacks. These episodes consisted of abrupt-onset frontal and periorbital pain associated with visual aura and responded well to supplemental oxygen and prednisone tapers. The patient had no known allergies. Medications included atorvastatin 20 mg daily, levothyroxine 75 mcg daily, and losartan 50 mg BID. Occupation and location of residence were unremarkable. The patient was Caucasian. There was no family history of balance abnormalities or tremor.

After initial presentation, medical management of the pre-existing ET was limited to gabapentin trial, as the patient previously did not tolerate primidone and has borderline bradycardia, limiting the use of propranolol. However, despite pharmacologic management, the patient’s most disabling symptom remained the essential tremor, therefore Cala Trio therapy was initiated. The device was worn intermittently on the left wrist according to manufacturer instructions between June 10 and June 28, 2024. The patient reported modest improvement in tremor severity during this initial treatment period. However, approximately 17 days after beginning Cala Trio therapy, on June 27^th^ 2024, the patient developed recurrent episodes of severe left-sided throat and neck pain consistent with glossopharyngeal neuralgia. He described brief, intense, burning pain localized to the left submandibular region that lasted approximately one minute and recurred every few minutes throughout the day. These episodes were reliably triggered by swallowing liquids and coughing and were associated with worsening hoarseness. The patient denied dysphagia or postnasal drip. Notably, both the wrist receiving stimulation and the neuralgia symptoms were left-sided. The patient reported that the pain characteristics were identical to a previous episode of glossopharyngeal neuralgia that had occurred approximately two years earlier, although symptoms had been quiescent prior to Cala Trio initiation.

Two years prior, an ENT consult proposed GERD as the mechanism. Repeat workup by ENT, following the Cala Trio-related event in 2024, ruled out any obvious laryngeal malformations on laryngoscopy. Esophagram showed findings of esophageal dysmotility with moderate gastroesophageal reflux, with an otherwise unremarkable examination. MRI imaging revealed volume loss and concordant ventricular enlargement with mild white matter T2 FLAIR hyperintensity most likely secondary to chronic small vessel disease. No findings of ischemic infarct, hemorrhage, or mass effect with no abnormal intracranial enhancement. There were no abnormalities along the posterior central skull base, jugular foramen, or hypoglossal canal. Prednisone 40 mg daily and tizanidine 4 mg at bedtime was prescribed with minimal relief.

The patient discontinued Cala Trio on June 28, 2024, after which the neuralgia gradually improved and resolved over the subsequent several days. To assess reproducibility, the patient restarted the device on the left wrist from June 30 through July 20, 2024. During the second trial, symptoms recurred with the same clinical characteristics as the initial episode, including left-sided burning submandibular pain triggered by speaking and swallowing and associated with hoarseness. Because of recurrent symptoms, Cala Trio was again discontinued. Following cessation of Cala Trio use, the neuralgia symptoms resolved. The patient did not trial the device on the contralateral wrist.

At subsequent follow-up, the patient reported intermittent recurrence of symptoms consistent with glossopharyngeal neuralgia despite remaining off Cala Trio therapy. These episodes occurred in late April 2025 and again during deep brain stimulation (DBS) screening evaluations on May 4–5, 2025. The recurrent episodes were qualitatively identical to prior events but were less severe overall, occurring less frequently and resolving more quickly. The patient described the attacks as still intensely painful when present. Although he had not routinely taken carbamazepine following the original episode, he used carbamazepine on two occasions during the May 2025 recurrence and reported symptomatic improvement. These later episodes suggest an underlying predisposition to glossopharyngeal neuralgia independent of Cala Trio use, while the temporal association, symptom exacerbation, and positive rechallenge observed during device therapy continue to support a potential role for peripheral neuromodulation as a trigger for symptom recurrence in this patient.

## Discussion

This case describes a 72-year-old man with longstanding ET and newly diagnosed PD who developed paroxysmal throat pain consistent with history of a self-resolved bout of GPN that was exacerbated within 17 days of initiating Cala Trio therapy. Structural, compressive, and inflammatory etiologies were excluded through laryngoscopy and neuroimaging. Although esophageal dysmotility and reflux were present, these findings were chronic and insufficient to explain the abrupt onset and severity of symptoms, and most notably, the resolution of neuralgic episodes after discontinuing the device. Taken together, the close temporal relationship and reversibility strongly suggest a stimulation-associated phenomenon, possibly in an at-risk individual. To our knowledge, exacerbation of GPN has not previously been reported as an adverse effect of TAPS in any published studies, case reports, FDA documentation, or post-market surveillance data or international regulatory agencies.

GPN is characterized by brief, stabbing attacks of pain in the posterior tongue, pharynx, or deep ear, typically triggered by swallowing, coughing, or speaking [24]. The glossopharyngeal nerve conveys somatic and visceral sensory input to the nucleus tractus solitarius and contributes motor fibers from the nucleus ambiguus. The prevailing pathophysiologic model involves hyperexcitability at the root entry zone of the nerve, where focal demyelination and ephaptic transmission are believed to facilitate abnormal cross-excitation between these adjacent fibers [25,26,27,28]. Functional instability within dorsal medullary circuits may further amplify paroxysmal discharges. Even in the absence of demonstrable vascular compression, abnormal central excitability may predispose susceptible individuals to episodic cranial neuropathic pain [24].

**Figure 1:**
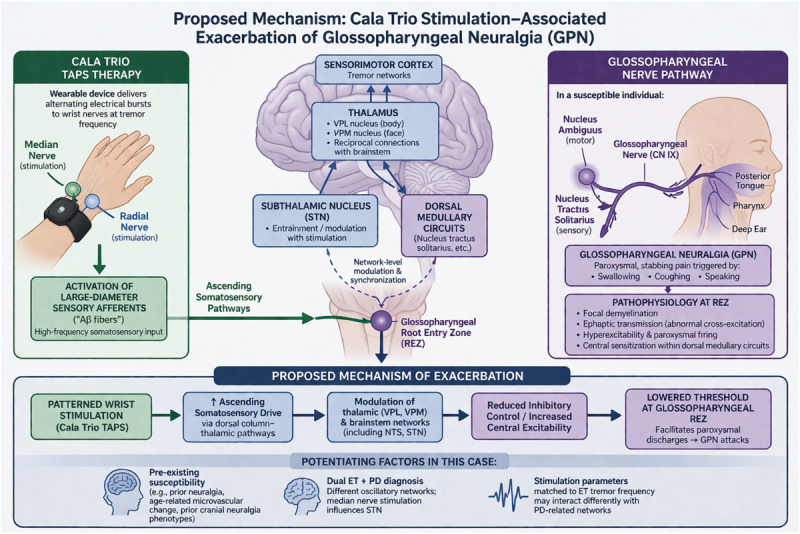
Proposed mechanism of TAPS-associated exacerbation of glossopharyngeal neuralgia.

The Cala system measures the individual’s tremor frequency and then delivers alternating electrical bursts to the median and radial nerves at the same frequency [21]. The therapeutic premise is that activation of large-diameter sensory afferents can modulate central tremor networks by disrupting synchronized abnormal signals through a process similar to deep brain stimulation. Prior neurophysiologic work has demonstrated that median nerve stimulation is known to generate high-frequency oscillatory responses in sensory thalamic nuclei, entraining neuronal firing patterns, and evoked responses have also been observed in the subthalamic nucleus [22]. These findings support the concept that peripheral afferent stimulation is not confined to spinal reflex arcs but influences distributed subcortical networks, including nodes involved in tremor generation. While the therapeutic target of TAPS is the cerebello-thalamo-cortical loop, the broader neuroanatomical consequences of patterned afferent input remain incompletely defined. Ascending somatosensory pathways traverse brainstem relay nuclei before reaching the thalamus, and reciprocal connections link thalamic nuclei with medullary structures. Repetitive, tremor-synchronized afferent stimulation could therefore modify central sensory gain or promote network-level synchronization extending beyond intended tremor circuits into brainstem nuclei involved in cranial nerve integration (Figure 1).

An additional mechanistic framework is informed by a reported case of GPN triggered by non-noxious stimuli at distant cephalic and extracephalic sites, including limb stimulation [29]. In this case study, Berg et al. discovered that light tactile input outside the glossopharyngeal territory provoked neuralgic attacks, and positron emission tomography demonstrated activation within the upper brainstem during extracephalic triggering. The authors proposed that low-threshold mechanosensory afferents, via dorsal column–thalamic projections and descending brainstem modulatory systems, may facilitate nociceptive nuclei when inhibitory control is impaired. In the present case, patterned stimulation of large-diameter afferents at the wrist from the Cala Trio device may have functioned analogously to these extracephalic triggers. If a pre-existing susceptibility existed at the glossopharyngeal root entry zone, whether related to age-associated microvascular change, prior inflammatory irritation, or prior cranial neuralgia and cluster headache phenotypes like seen in this patient, altered central excitability could have lowered the threshold for paroxysmal firing within glossopharyngeal pathways (Figure 1).

An additional consideration is the patient’s dual diagnosis of ET and PD. Clinical trials evaluating Cala devices primarily enrolled individuals with isolated ET and demonstrated favorable safety profiles without reports of cranial neuropathic pain [16,17,18,19,20,21]. Parkinsonian tremor, however, arises from partially distinct oscillatory mechanisms involving basal ganglia-thalamo-brainstem circuits [30,31]. Because median nerve stimulation has been shown to influence subthalamic activity [22], stimulation parameters optimized for ET frequency may interact differently in the context of PD-related network dynamics. It is plausible that coexisting basal ganglia pathology altered the system’s response to patterned sensory input, thereby facilitating unintended modulation of adjacent brainstem pathways.

Overall, this case represents the first report of GPN exacerbation secondary to Cala Trio treatment. Although causality cannot be definitively established from a single case, the rapid resolution of symptoms following cessation and recurrence with a second trial argue against structural injury and instead favor a reversible neurophysiologic mechanism. This report expands safety considerations of TAPS therapy and underscores the importance of monitoring for atypical neurologic symptoms, particularly in patients with mixed tremor phenotypes or prior cranial neuralgia. As peripheral neuromodulation technologies evolve, further investigation into their effects on brainstem sensory nuclei and cranial nerve circuits will be essential to define both therapeutic mechanisms and rare adverse events.
