# Correction: A Case of Cala Trio-Induced Glossopharyngeal Neuralgia in a Patient with Essential Tremor and Idiopathic Parkinson’s Disease

**DOI:** 10.5334/tohm.1258

**Published:** 2026-07-09

**Authors:** Moriah R. Arnold, Amie L. Hiller

**Affiliations:** 1 Medical Scientist Training Program, Oregon Health and Science University, Portland, OR, USA; 2 Neurology Department, Oregon Health and Science University, Portland, OR, USA; 3 VA PADRECC – Portland Health Care System, Portland, OR, USA

**Keywords:** Essential Tremor, Transcutaneous afferent patterned stimulation, Glossopharyngeal neuralgia, Neuromodulation, Parkinson’s Disease

## Abstract

This article details a correction to Arnold, M.R. and Hiller, A.L. (2026) ‘A Case of Cala Trio-Induced Glossopharyngeal Neuralgia in a Patient with Essential Tremor and Idiopathic Parkinson’s Disease’, *Tremor and Other Hyperkinetic Movements*, 16(1), p. 41. Available at: https://doi.org/10.5334/tohm.1224.

## Correction

The original Figure 1 in “A Case of Cala Trio-Induced Glossopharyngeal Neuralgia in a Patient with Essential Tremor and Idiopathic Parkinson’s Disease” by Arnold and Hiller unintentionally included an illustration of a hand with six digits [1]. The figure should have featured a hand with five digits.

**Figure 1:**
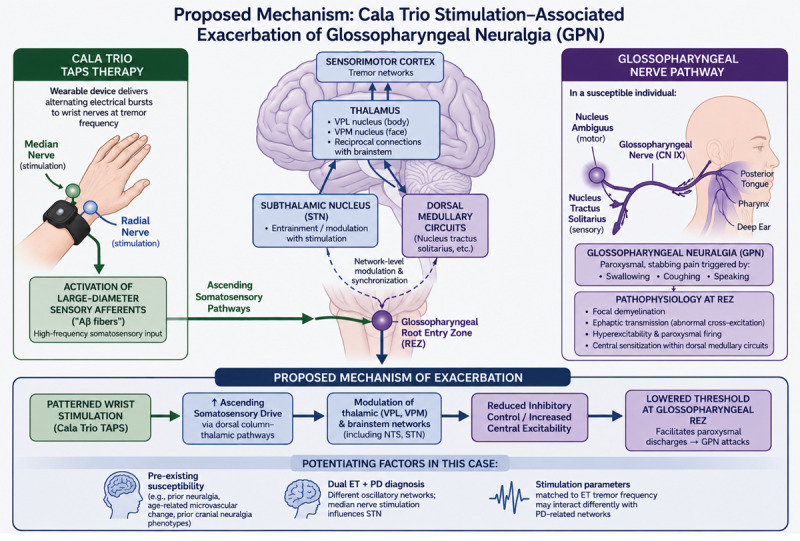
Proposed mechanism of TAPS-associated exacerbation of glossopharyngeal neuralgia.

The corrected Figure 1 is featured above:
